# The MASTL-ENSA-PP2A/B55 axis modulates cisplatin resistance in oral squamous cell carcinoma

**DOI:** 10.3389/fcell.2022.904719

**Published:** 2022-09-28

**Authors:** Odjo G. Gouttia, Jing Zhao, Yanqiu Li, Mackenzie J. Zwiener, Ling Wang, Gregory G. Oakley, Aimin Peng

**Affiliations:** Department of Oral Biology, University of Nebraska Medical Center, Lincoln, NE, United States

**Keywords:** MASTL, greatwall kinase, DNA damage, oral cancer, cisplatin, OSCC

## Abstract

Platinum-based chemotherapy is the standard first-line treatment for oral squamous cell carcinoma (OSCC) that is inoperable, recurrent, or metastatic. Platinum sensitivity is a major determinant of patient survival in advanced OSCC. Here, we investigated the involvement of MASTL, a cell cycle kinase that mediates ENSA/ARPP19 phosphorylation and PP2A/B55 inhibition, in OSCC therapy. Interestingly, upregulation of MASTL and ENSA/ARPP19, and downregulation of PP2A/B55, were common in OSCC. MASTL expression was in association with poor patient survival. In established OSCC cell lines, upregulation of MASTL and ENSA, and downregulation of B55 genes, correlated with cisplatin resistance. We further confirmed that stable expression of MASTL in OSCC cells promoted cell survival and proliferation under cisplatin treatment, in an ENSA-dependent manner. Conversely, deletion of MASTL or ENSA, or overexpression of B55α, sensitized cisplatin response, consistent with increased DNA damage accumulation, signaling, and caspase activation. Moreover, GKI-1, the first-in-class small molecule inhibitor of MASTL kinase, phenocopied MASTL depletion in enhancing the outcome of cisplatin treatment in OSCC cells, at a dose substantially lower than that needed to disrupt mitotic entry. Finally, GKI-1 exhibited promising efficacy in a mouse tumor xenograft model, in conjunction with cisplatin therapy.

## Introduction

Oral cancer is the sixth most common cancer worldwide. In the United States, approximately 50,000 new oral cancer cases are diagnosed each year. Over 90% of oral cancer cases are oral squamous cell carcinomas (OSCC) arising from the oral epithelium. Compared to many other major types of cancer, the treatment option and overall survival for oral cancer has not markedly improved over the last 3 decades. While OSCC at early stages can be cured largely by surgery alone, the majority of OSCC cases are diagnosed at later stages (III and IV), and are typically treated with surgery and external radiotherapy, in combination with chemotherapeutic agents. Unfortunately, the prognosis for advanced OSCC, especially those not associated with human papilloma virus (HPV), remains poor. Thus, a major challenge for oral cancer treatment lies in the intrinsic or acquired mechanisms that render tumor cells resistance to radiation and chemotherapy (Casiglia and Woo, 2001; Zygogianni et al., 2011; Rivera, 2015; Ali et al., 2017).

Cisplatin (cis-diaminedichloroplatinum) and other platinum analogs are widely used in the treatment of solid tumors. Cisplatin manifests its cytotoxic effect by inducing inter- and intrastrand crosslinked DNA adducts. These forms of DNA damage disrupt DNA metabolism, especially DNA replication and transcription, thereby suppressing cell proliferation and triggering cell death (Dasari and Tchounwou, 2014; Gau et al., 2019). Importantly, our cells possess a collection of complex and evolutionarily conserved mechanisms to sense, and respond to, the induction of DNA damage (Sancar et al., 2004; Jackson and Bartek, 2009; Lord and Ashworth, 2012). DNA repair is a core element of the DNA damage response (DDR). To date, numerous lesion-specific DNA repair pathways have been characterized, with over 100 DNA repair genes identified. For example, multiple DNA repair mechanisms, including nucleotide excision repair, mismatch repair, double strand break repair and interstrand crosslink repair, have been implicated in cisplatin-induced DNA damage (Sancar et al., 2004). Furthermore, DNA damage activates a signaling cascade, composed of ATM (ataxia telangiectasia mutated), ATR (ATM and Rad3-related), CHK1 (checkpoint kinase 1), CHK2 (checkpoint kinase 2) kinases and other factors, to engage DNA damage checkpoints and arrest cell cycle progression. Ultimately, if the level of DNA damage overwhelms cellular repair capability, cells will be eliminated through caspase-3-dependent apoptosis and other cell death pathways (Melo and Toczyski, 2002; Shiloh, 2003).

Recent studies in various model systems characterized microtubule-associated serine/threonine kinase like (MASTL, also known as Greatwall) as an important regulator of mitosis. MASTL is activated during mitotic entry via CDK1-mediated phosphorylation, and the kinase activity of MASTL is required for mitotic progression (Yu et al., 2004; Yu et al., 2006; Archambault et al., 2007; Castilho et al., 2009; Peng and Maller, 2010; Voets and Wolthuis, 2010; Vigneron et al., 2011; Blake-Hodek et al., 2012). It has been revealed that, upon activation, MASTL phosphorylates α-endosulfine (ENSA) and cyclic AMP-regulated 19 kDa phosphoprotein (ARPP19). Phosphorylated ENSA and ARPP19 then bind and inhibit PP2A/B55 (protein phosphatase 2A with a B55 targeting subunit) which is the principal phosphatase holoenzyme that dephosphorylates substrates of CDK1 (Castilho et al., 2009; Mochida et al., 2009; Vigneron et al., 2009; Gharbi-Ayachi et al., 2010; Mochida et al., 2010). Furthermore, we reported that MASTL modulates DNA damage signaling and facilitates cell cycle recovery from the G2/M DNA damage checkpoint in *Xenopus* egg extracts (Peng et al., 2010a; Peng et al., 2011a). Interestingly, we and other groups showed MASTL upregulation in multiple types of cancer, in association with aggressive clinicopathological features (Wang et al., 2014a; Vera et al., 2015; Sun et al., 2017; Tian et al., 2017; Zhuge et al., 2017; Alvarez-Fernandez et al., 2018; Rogers et al., 2018; Uppada et al., 2018; Fatima et al., 2021). In this study, we delineated the MASTL-ENSA/ARPP19-PP2A/B55 pathway as an important determinant of cisplatin resistance and clinical treatment outcome in OSCC, and validated MASTL inhibition as a potentially valuable therapeutic strategy in combinatorial cancer therapy with cisplatin.

## Materials and methods

### Cell culture and treatment

Human oral squamous-cell carcinoma cell line SCC38 (UM-SCC-38), as characterized in previous studies (Brenner et al., 2010; Wang et al., 2012; Khanh et al., 2016), were maintained in Dulbecco’s modified Eagle medium (DMEM, Sigma) supplemented with 10% fetal bovine serum and 1% antibiotics. Human tongue squamous-cell carcinoma Cal27 cells were purchased from ATCC, and maintained in DMEM. CFP-tagged MASTL was constructed to pLZBob, a retroviral vector provided by Dr. James Wahl at the University of Nebraska Medical Center. The cell population stably expressing CFP-MASTL were isolated by G418 selection. Cisplatin and nocodazole were obtained from Sigma, and used for cell treatment as specified in the experiments. GKI-1 was characterized in a previous study (Ocasio et al., 2016). SiRNA targeting MASTL or ENSA was purchased from Integrated DNA Technologies (IDT). These siRNAs were transfected into cells with Lipofectamine RNAiMAX Transfection Reagent, using the protocol recommended by the manufacturer. HA-PPP2R2A (B55α) expression vector, as described in our previous study (Wang et al., 2018), was transfected into cells with Lipofectamine, using the protocol recommended by the manufacturer.

### Cell viability and soft agar growth assays

To measure cell growth and sensitivity to cisplatin, cells were treated without or with MASTL or ENSA siRNA for 1 day, and then incubated in cisplatin, with or without GKI-1, at the indicated concentrations for 1–4 days. The numbers of viable cells were counted using a hemocytometer. The concentration of cisplatin that inhibited 50% cell growth (IC50) was calculated as in our previous study (Wang et al., 2012). Briefly, cells were treated with various concentrations of cisplatin, ranging from 1–10 μM. Cell numbers were counted 2 days post treatment. For anchorage-independent cell growth, cells were grown in 0.3% agar on a cushion of 0.6% agar in 35-mm plates, as described previously (Peng et al., 2010b).

### Database analysis

The gene expression data for MASTL, ENSA, ARPP19, PPP2R2A and PPP2R2B were obtained from previous studies (Cromer et al., 2004; Ginos et al., 2004; Sengupta et al., 2006; Ye et al., 2008; Estilo et al., 2009; Peng et al., 2011b). The box plot diagrams were generated using Microsoft Excel, with the medium value of the control group set as zero. For the OSCC cell line data analyses, gene expression profiles (GSE36133) were obtained from the Cancer Cell Line Encyclopedia (CCLE) project. The OSCC IC50 data for cisplatin were downloaded from the Genomics of Drug Sensitivity in Cancer (GDSC) database.

### Immunoblotting and immunohistochemistry

Sodium dodecyl-sulfate polyacrylamide gel electrophoresis (SDS-PAGE) and immunoblotting was performed as previously described (Ren et al., 2017). The following primary antibodies were used: anti-MASTL (MABT372, Millipore), anti-active-caspase 3 (ab47131, Abcam), anti-phospho-CHK2 (Thr-68, #2661, Cell Signaling Technology), anti-CHK2 (#6334, Cell Signaling Technology), anti-cleaved PARP1 (#9541, Cell Signaling Technology), anti-ENSA/ARPP19 (ab180513, Abcam), anti-γ-H2AX (Ser-139, SC-517348, Santa Cruz Biotechnology), anti-H2B (#12364, Cell Signaling Technology), anti-H2AX (SC-517336, Santa Cruz Biotechnology), anti-PPP2R2A (B55α, GTX111128, Genetex), anti-SMC1 phospho-Ser-957 (A300-045, Bethyl Laboratories), anti-α-tubulin (from Dr. James Wahl, as in (Wang et al., 2021)). Immunohistochemistry analysis was performed as in a previous study (Wang et al., 2014a). As described in (Rector et al., 2016), OSCC tissues were obtained from the University of Nebraska Medical Center College of Medicine Department of Pathology and Microbiology, with applicable, biographical data and disease-specific medical history obtained from the UNMC College of Medicine Department of Otolaryngology/Head & Neck Surgery. Institutional Review Board (IRB) approval was obtained for the performed experiments. The slides were deparaffinized, and autoclaved for antigen retrieval. Slides were treated with 3% hydrogen peroxide solution, and blocked with 10% normal goat serum in PBS, prior to incubation in primary antibody (anti-MASTL, MABT372, Millipore). Bound antibody was detected with a streptavidin-biotin system suing DAB substrate. Hematoxylin counterstain was performed.

### Mouse tumor studies

Athymic nude mice were purchased from the Jackson Laboratory, and housed at the UNMC College of Dentistry animal facility. SCC38 cells were implanted into 6-week old mice by a single subcutaneous injection (5 × 10^5^ cells in 100 microliters of sterile PBS). To test the tumor response to chemotherapy, once the tumor size reached 100 mm^3^, cisplatin (5 mg/kg mouse), with or without GKI-1 (10 mg/kg mouse) were administered intraperitoneally, using a three dose schedule with 3-day intervals. Twenty days after the initial treatment, the mice were euthanized, and tumors were removed and weighed. Tumor lysates were collected for immunoblotting analysis, as described in previous study (Wang et al., 2014a).

### Statistical analysis

Statistical analyses were performed in cell viability assays and in the tumor weight measurements. Briefly, data were analyzed using an unpaired 2-tailed Student’s t test to determine the statistical significance. A *p*-value less than 0.05 is considered as significant. The association between cisplatin IC50 and gene expression level of MASTL pathway was analyzed by Pearson correlation coefficient. The survival probability in MASTL high or low groups were calculated using the Kaplan-Meier method and compared using the log-rank test.

## Results

### MASTL-ENSA/ARPP19-B55 expression was dysregulated in OSCC

**FIGURE 1:**
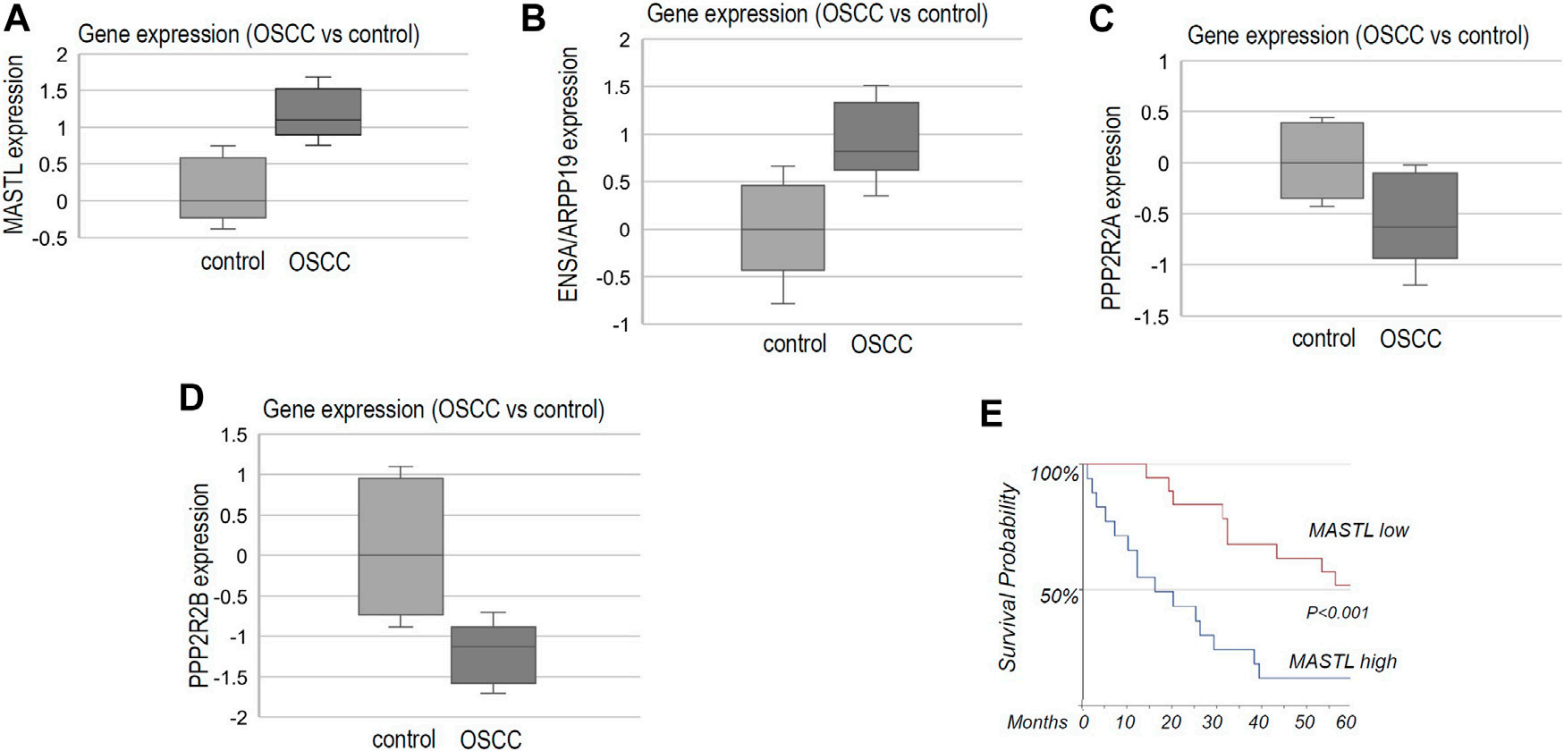
The MASTL-ENSA/ARPP19-PP2A/B55 pathway is dysregulated in OSCC, in association with patient survival. **(A)** The gene expression data for MASTL, ENSA, ARPP19, PPP2R2A and PPP2R2B were obtained, as described in Materials and Methods. The expression level of MASTL in control (normal, *n* = 44) or OSCC tumor tissues (*n* = 114) was shown in the box plot diagram (in log2 scale). **(B)** The expression level of ENSA + ARPP19 in control (normal, *n* = 53) or OSCC tumor tissues (*n* = 137) was shown in the box plot diagram (in log2 scale). **(C)** The expression level of PPP2R2A in control (normal, *n* = 39) or OSCC tumor tissues (*n* = 132) was shown in the box plot diagram (in log2 scale). **(D)** The expression level of PPP2R2B in control (normal, *n* = 48) or OSCC tumor tissues (*n* = 89) was shown in the box plot diagram (in log2 scale). **(E)** As described in Materials and Methods, OSCC tumor samples were obtained for 35 HPV- oral patients who were treated at the University of Nebraska Medical Center. Immunohistochemistry was performed to detect MASTL expression. The survival probability was shown for patients with high or low levels of MASTL expression.

By surveying previous studies that profiled gene expression in OSCC (Cromer et al., 2004; Ginos et al., 2004; Sengupta et al., 2006; Ye et al., 2008; Estilo et al., 2009; Peng et al., 2011b), we found significantly elevated expression of MASTL in tumor samples, in comparison to normal controls (Figure 1A). Moreover, the combined expression of ENSA and ARPP19 exhibited a similar pattern of upregulation in OSCC (Figure 1B). In contrast, PPP2R2A and PPP2R2B, two B55 subunits, were largely suppressed in OSCC (Figures 1C,D). We then evaluated MASTL expression in HPV- oral cancer patients treated at the UNMC Department of Otolaryngology/Head & Neck Surgery. Compelling to us, the study revealed a highly significant correlation between MASTL upregulation and poor patient survival (Figure 1E).

### The MASTL-ENSA pathway modulated the cisplatin response in OSCC

**FIGURE 2:**
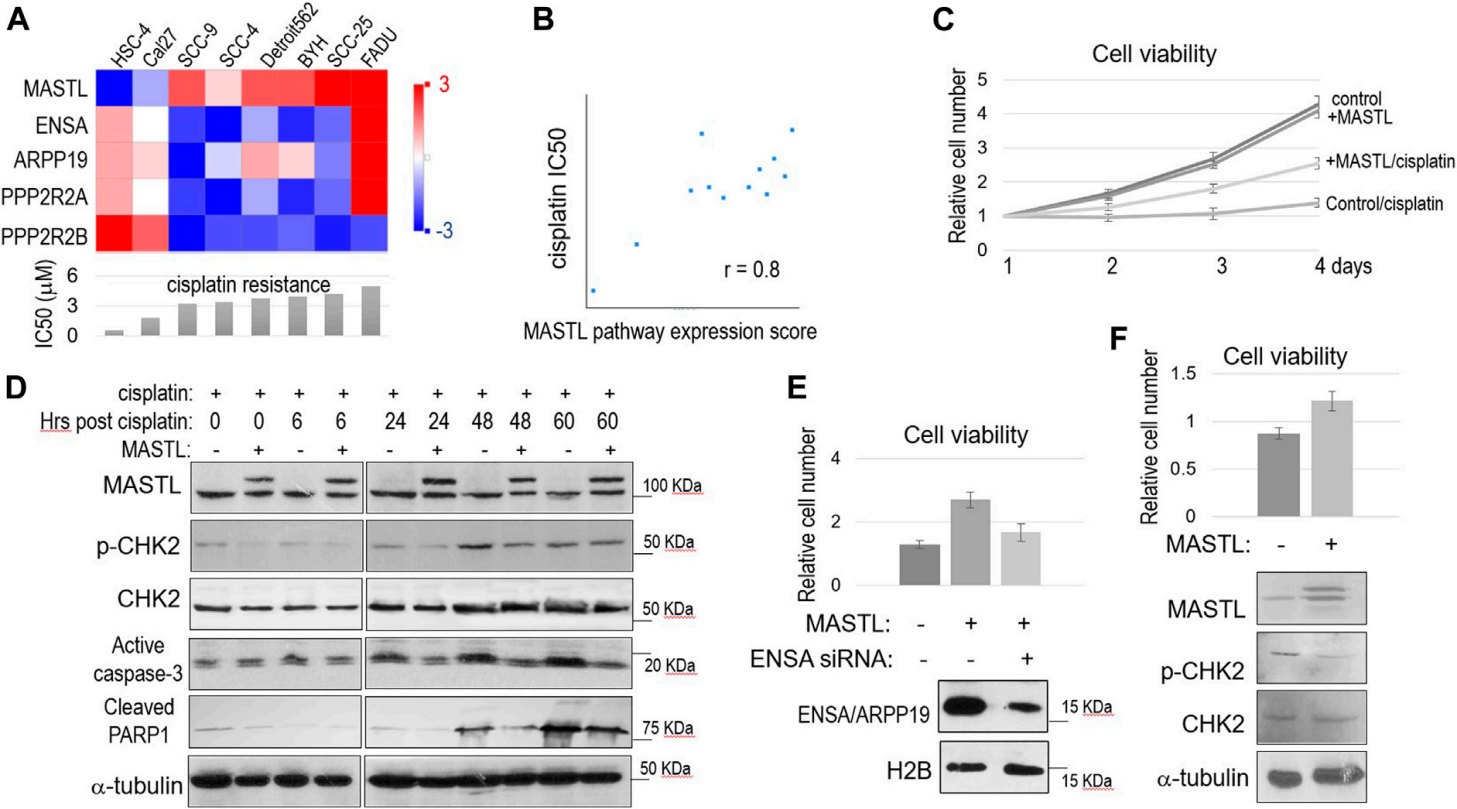
The MASTL-ENSA/ARPP19-PP2A/B55 pathway mediates cisplatin resistance in OSCC. **(A)** The expression levels of MASTL, ENSA, ARPP19, PPP2R2A, and PPP2R2B in various OSCC cell lines were obtained, and shown in the heatmap graph, as described in Materials and Methods. The IC50 of cisplatin was shown in the below panel. **(B)** The expression levels of MASTL, ENSA, ARPP19, PPP2R2A, and PPP2R2B in OSCC cell lines were obtained as in panel **(A)**. The MASTL pathway expression score was calculated by MASTL + ENSA + ARPP19-PPP2R2A-PPP2R2B (log2 values), and correlated with cisplatin IC50 values, as described in Materials and Methods. **(C)** Cell viability assay was performed as in Materials and Methods. SCC38 cells with or without stable expression of CFP-MASTL were incubated with cisplatin (3.3 μM) from day 1. The cell numbers at days 2–4 were normalized to that at day 1 (untreated). The mean values and standard derivations, from three independent experiments, were shown. **(D)** SCC38 cells without or with stably expression of CFP-MASTL were treated with cisplatin (5 μM) for 2 h, and allowed for recovery for the indicated numbers of hours. Cells were harvested and analyzed by immunoblotting, using the indicated antibodies. **(E)** SCC38 cells with or without CFP-MASTL expression or ENSA siRNA-treatment were cultured in the presence of cisplatin (3.3 μM). After 3 days of cisplatin treatment, cell viability was measured, as in panel **(C)**. Mean values and standard deviations were calculated from three independent experiments, and shown in the upper panel. The effect of ENSA knockdown was shown by immunoblotting, in the lower panel, using antibodies that recognize H2B and both ENSA/ARPP19. **(F)** Cal27 cells transfected with control GFP or GFP-MASTL expression vectors were treated with cisplatin (3.3 μM). The cell viability after 2 days incubation was determined, and normalized to that of pre-treatment. The mean values and standard deviations were calculated from three independent experiments, and shown in the upper panel. Cells were analyzed by immunoblotting using the indicated antibodies, and shown in the lower panel.

We analyzed the Cancer Cell Line Encyclopedia (CCLE) database for a potential connection between cisplatin resistance and the expression of MASTL and its downstream factors. In an array of OSCC cell lines with different levels of cisplatin resistance, a higher IC50 of cisplatin was in general correlation with higher expression of MASTL, ENSA and ARPP19, but with lower PPP2R2A (B55α) and PPP2R2B (B55β) expression (Figure 2A). We then showed that the overall expression scores of the MASTL pathway, as calculated by log2 (MASTL) + log2 (ENSA) + log2 (ARPP19)—log2 (PPP2R2A)—log2 (PPP2R2B), are in strong positive association with cisplatin resistance in the collection of OSCC cell lines (Figure 2B).

We sought to investigate the functional impact of MASTL upregulation in OSCC cells. SCC38 was selected for the study, as this HPV- OSCC cell line was previously characterized to be highly resistant to cisplatin, consistent with the poor clinical treatment outcome of the patient from whom this cell line was derived (Wang et al., 2012). We generated SCC38 cells that stably expressed recombinant MASTL to a level similar to endogenous MASTL. Compared to the control SCC38 cells, SCC38 harboring 2-fold MASTL upregulation exhibited a very strong proliferative advantage in the presence of cisplatin (Figure 2C). In comparison, both cell lines showed near identical rates of cell proliferation without cisplatin, suggesting specific function of MASTL in the cellular response to cisplatin (Figure 2C). Moreover, MASTL overexpression reduced DNA damage signaling, as shown by CHK2 phosphorylation, and the induction of cell death, as indicated by active caspase-3 and PARP1 cleavage, in response to cisplatin (Figure 2D). The function of MASTL in conferring cisplatin resistance was mediated by ENSA, as ENSA knockdown reversed the effect of MASTL expression (Figure 2E). We determined that ENSA was more dominantly expressed in these cells, compared to ARPP19, because knockdown of ENSA significantly reduced the total expression of ENSA and ARPP19 (Figure 2E). Finally, we sought to confirm the effect of MASTL expression in Cal27 cells that featured relatively low endogenous MASTL expression and high cisplatin sensitivity (Figure 2A). Expression of exogenous MASTL in Cal27 increased cell viability and reduced CHK2 phosphorylation, in the presence of cisplatin (Figure 2F), consistent with the observations in SCC38.

### Depletion of MASTL or ENSA, or upregulation of B55, enhanced the cisplatin response

**FIGURE 3:**
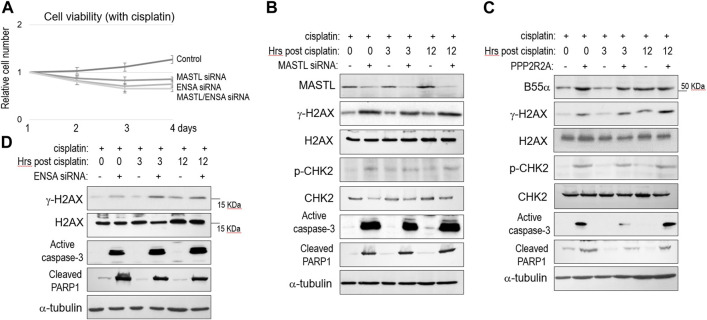
The MASTL-ENSA/ARPP19-PP2A/B55 pathway modulates the cisplatin response in OSCC. **(A)** Cell viability assay was performed as in Materials and Methods. SCC38 cells with or without MASTL or ENSA siRNA were incubated with cisplatin (3.3 μM) from day 1. The cell numbers at days 2–4 were normalized to that at day 1 (untreated). The mean values and standard derivations, from three independent experiments, were shown. **(B)** SCC38 cells with or without MASTL siRNA were treated with cisplatin (5 μM) for 2 h, and allowed for recovery for the indicated numbers of hours. Cells were harvested and analyzed by immunoblotting, using the indicated antibodies. **(C)** SCC38 cells with or without HA-PPP2R2A expression were treated with cisplatin (5 μM) for 2 h, and allowed for recovery for the indicated numbers of hours. Cells were harvested and analyzed by immunoblotting, using the indicated antibodies. **(D)** SCC38 cells with or without ENSA siRNA were treated with cisplatin (5 μM) for 2 h, and allowed for recovery for the indicated numbers of hours. Cells were harvested and analyzed by immunoblotting, using the indicated antibodies.

To the contrary of MASTL overexpression, partial depletion of MASTL expression using siRNA reduced SCC38 cell viability after cisplatin treatment (Figure 3A). Depletion of ENSA exhibited a similar outcome, whereas simultaneous depletion of MASTL and ENSA showed no additive effect, in comparison with single depletion (Figure 3A). Thus, MASTL and ENSA acted in the same pathway to mediate cisplatin resistance. At the molecular level, MASTL depletion led to enhanced DNA damage (γ-H2AX), signaling (CHK2 phosphorylation), and cell death (caspase-3 activation and PARP1 cleavage, Figure 3B). Similar molecular events were observed in SCC38 cells with upregulation of B55α (Figure 3C), indicating that the function of MASTL was indeed mediated by PP2A/B55 suppression through ENSA/ARPP19. To this end, we confirmed also that ENSA depletion enhanced H2AX phosphorylation, caspase-3 activation, and PARP1 cleavage post-cisplatin (Figure 3D).

### MASTL inhibition using GKI-1 overcame cisplatin resistance in OSCC

**FIGURE 4:**
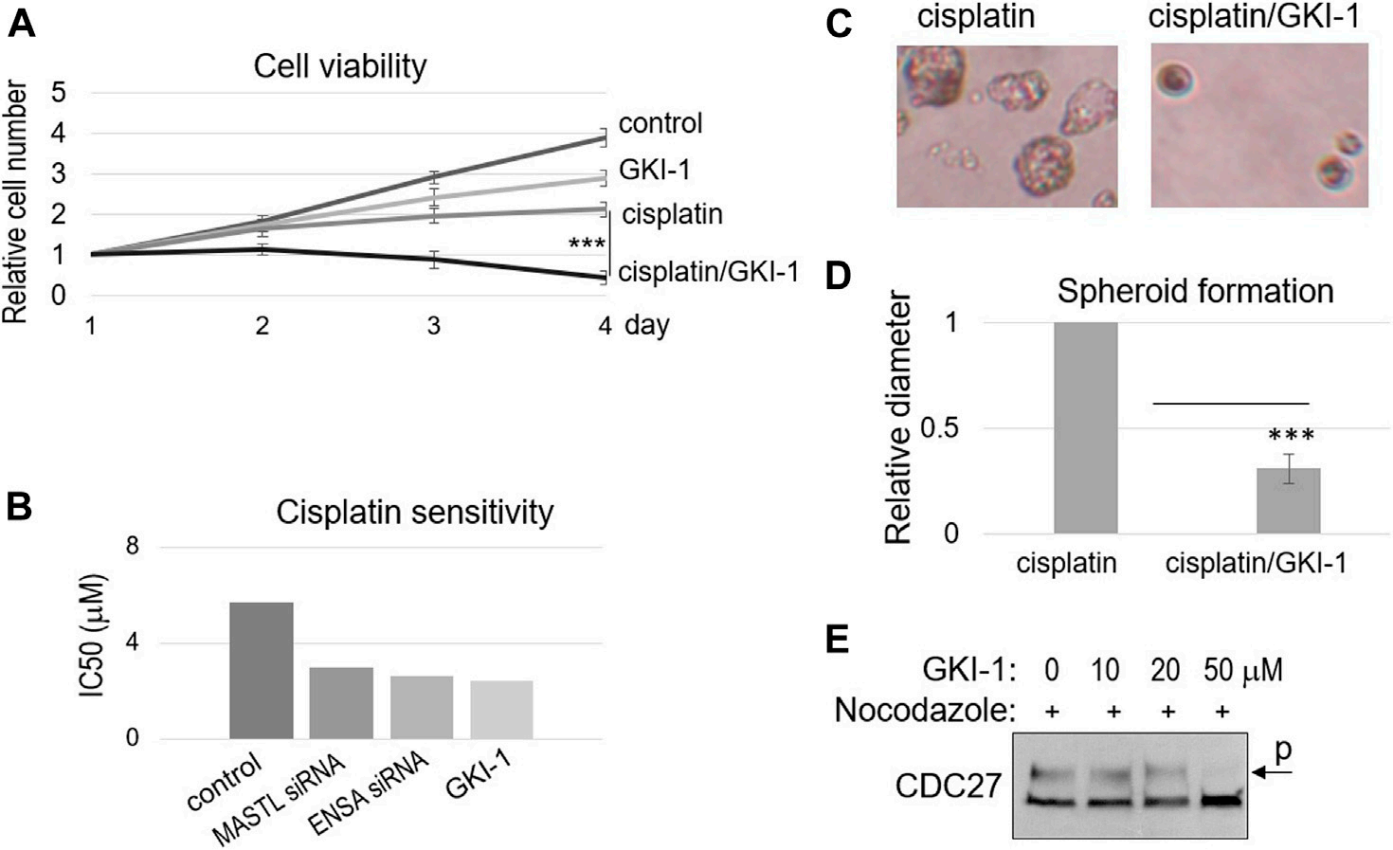
GKI-1 sensitizes OSCC cells to cisplatin. **(A)** SCC38 cells were treated with or without GKI-1 (10 μM) and cisplatin (3.3 μM) for 3 days. The cell viability was studied as described in Materials and Methods. The cell numbers at days 2–4 were normalized to that at day 1 (untreated). The mean values and standard derivations, from three independent experiments, were shown. **(B)** SCC38 cells were treated with MASTL siRNA, ENSA siRNA, or GKI-1, as indicated. IC50 of cisplatin was determined, as described in Materials and Methods, and shown. **(C,D)** SCC38 cells treated with GKI-1 and cisplatin were cultured in soft agar for 14 days. Representative spheroid formation was shown in panel C, and average diameters were shown in panel D (N > 20, *p* < 0.001). **(E)** SCC38 cells were treated with nocodazole (1 μg/ml) for 12 h, so that cells entered mitosis will be arrested/trapped in mitosis. The cells were also treated with various concentrations of GKI-1, as indicated, to prevent mitotic entry. Cells were harvested and analyzed by immunoblotting for CDC27. The phosphorylation of CDC27 retards its gel migration, and is commonly used as a marker of mitosis.

Ocasio et al. discovered the first small molecule inhibitor of MASTL kinase, named GKI-1 (Greatwall kinase inhibitor-1) (Ocasio et al., 2016). That previous study confirmed the cellular efficacy of GKI-1, including the reduction of ENSA/ARPP19 phosphorylation (Ocasio et al., 2016). Interestingly, we showed that GKI-1 sensitized SCC38 cells to cisplatin (Figure 4A). GKI-1 alone moderately impacted cell proliferation, but a combination of GKI-1 and cisplatin synergistically suppressed SCC38 viability (Figure 4A). IC50 analysis showed comparable levels of cisplatin sensitization by GKI-1 and the depletion of MASTL or ENSA (Figure 4B). Moreover, the effect of GKI-1/cisplatin combination was confirmed by spheroid formation, which was much more efficiently hindered by the combination than by cisplatin alone (Figures 4C,D). Given the role of MASTL in mitotic progression, we also sought to determine the effect of GKI-1 in preventing mitosis. Only at a high concentration (50 μM) was GKI-1 capable of blocking mitotic entry (Figure 4E). By comparison, cisplatin sensitization was readily achieved by GKI-1 at 10 μM.

**FIGURE 5:**
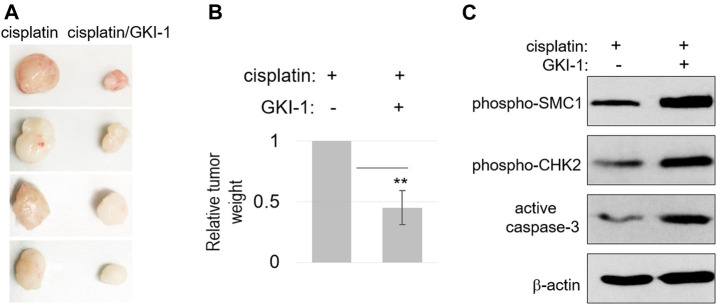
GKI-1 enhances the tumor response to cisplatin *in vivo*. **(A–C)** SCC38 xenograft tumor model was established, as described in Materials and Methods. Once tumor volume reached 100mm^3^, three doses of cisplatin and GKI-1 were administered Intraperitoneally with 3-day intervals. At the end of the study, tumors were excited and weighted. Tumor photos were shown in panel **(A)**. The mean values of tumor weight (*n* = 4), standard deviation, and power analysis (2-tailed t-test) were shown in panel **(B)**. Tumor analysis by immunoblotting was shown in panel **(C)**.

To further evaluate the therapeutic efficacy of GKI-1, we established a xenograft tumor model in mice, using SCC38 cells. These tumors were treated with either cisplatin alone or cisplatin/GKI-1. The combination treatment consistently resulted in more reduction of tumor volume (Figures 5A,B). We analyzed the tumor lysates and detected elevated levels of DNA damage signaling (CHK2 and SMC1 phosphorylation) and cell death (active caspase-3, Figure 5C).

## Discussion

We showed in the current study that MASTL and its downstream substrates ENSA and ARPP19 are frequently upregulated in OSCC, whereas B55 subunits of PP2A are downregulated. Thus, the MASTL-ENSA/ARPP19-PP2A/B55 pathway is of strong interest to the pathophysiology of OSCC. OSCC patients with high levels of MASTL expression suffered adverse treatment outcome, as indicated by shorter overall survival. Notably, MASTL upregulation has been observed also in breast, colon, and other types of cancer (Castro and Lorca, 2018; Marzec and Burgess, 2018; Fatima et al., 2020). In breast cancer, MASTL overexpression promoted chromosomal instability, and was correlated with disease progression and poor prognosis. Conversely, ablation of MASTL expression impaired the proliferation and metastasis of MDA-MB-231 breast cancer cells *in vitro* and *in vivo* (Vera et al., 2015; Zhuge et al., 2017; Alvarez-Fernandez et al., 2018; Rogers et al., 2018; Yoon et al., 2018). Thus, MASTL is likely to play a broad role in promoting tumor progression in various types of cancer, and the translational potential of MASTL targeting can be extended beyond OSCC.

During mitotic progression, MASTL functions by phosphorylating ENSA/ARPP19, which then inhibits PP2A/B55, preventing it from dephosphorylating CDK substrates. This mode of MASTL function is evolutionarily conserved, as a similar pathway has been reported in other vertebrate and invertebrate organisms, such as frog, fly, and yeast (Glover, 2012; Wang et al., 2014b; Vigneron et al., 2016; Castro and Lorca, 2018). It shall be noted, however, that additional, and distinct, mechanisms have been implicated for MASTL. For example, MASTL promoted AKT phosphorylation in MDA-MB-231 cells, in a manner that was not related to the decreased PP2A/B55 activity through ENSA/ARPP19 phosphorylation (Vera et al., 2015). Furthermore, MASTL was shown to promote cell contractility and motility independent of its kinase activity (Taskinen et al., 2020).

In this study, we highlighted the therapeutic potential of MASTL targeting in OSCC, in conjunction with cisplatin. Cisplatin and other platinum-based drugs are fundamental chemotherapeutics in the treatment of OSCC and other solid tumors. In advanced OSCC, the therapeutic response to cisplatin is a major determinant of treatment outcome and patient survival. Of a strong interest to us, analyses of data obtained in a panel of OSCC cell lines established a robust correlation between cisplatin resistance and the expression profiles of MASTL and its downstream factors. High levels of MASTL, ENSA, and ARPP19 expression, and low levels of PPP2R2A and PPP2R2B, were indicatives of cisplatin resistance. Among them, PPP2R2B and MASTL exhibited the highest predictive values. We believe that the role of MASTL in cisplatin response is consistent with previous studies that connected MASTL to the cell cycle recovery from DNA damage (Peng et al., 2010a; Peng et al., 2011a; Wang et al., 2014a; Wong et al., 2016). Indeed, MASTL overexpression in OSCC reduced cisplatin-induced DNA damage signaling and caspase activation, whereas MASTL depletion elevated the levels of DNA damage signaling and cell death. We presented further evidence to show that MASTL mediated cisplatin resistance via its downstream ENSA/ARPP19 and PP2A/B55. MASTL/ENSA double depletion did not further enhance cisplatin sensitivity over the single depletion; ENSA knockdown, or B55 expression, elicited similar effects in DNA damage signaling and caspase activation, as MASTL depletion. Taken together, our studies defined an important role of MASTL in conferring tumor cell resistance to cisplatin. We believe that this function of MASTL is mediated largely by its canonical downstream ENSA/ARPP19 phosphorylation and PP2A/B55 inhibition. Future studies are needed to reveal specific phospho-substrates of B55 that potentially mediate DNA repair, cell cycle progression, and cell death post cisplatin treatment.

Protein kinases are potentially druggable, and many of them have been extensively investigated as therapeutic targets in cancer (Bhullar et al., 2018; Cohen et al., 2021). For example, cell cycle kinases, such as cyclin-dependent kinases (CDK), polo-like kinases (PLK) and Aurora Kinases, are essential for cell proliferation. As a result, numerous pharmacological agents that inhibit these cell cycle kinases are either clinically approved for cancer treatment, or under clinical development toward FDA-approval (Taylor and Peters, 2008; Lapenna and Giordano, 2009; Otto and Sicinski, 2017). Along this line, MASTL is likely to yield translational potentials, given its involvement in cell cycle progression. Importantly, our study strongly indicated the therapeutic benefit of the combinatorial treatment composed of MASTL targeting and cisplatin. Using GKI-1, the first-in-class small molecule inhibitor of MASTL, we validated the synergistic effect between MASTL inhibition and cisplatin treatment. The results obtained in OSCC cells using GKI-1 were consistent with those observed with MASTL depletion. Intriguingly, the concentration of GKI-1 that conferred cisplatin sensitization was substantially lower than that required to block mitotic progression. This finding suggested a more promising application of MASTL targeting in cisplatin sensitization than in suppressing cell proliferation. A possible explanation for this differential dose requirement is that the kinase activity of MASTL is much lower in interphase than in mitosis, hence suppressing MASTL in interphase for cisplatin sensitization represents a more effective way of intervention. Finally, we confirmed the efficacy of GKI-1/cisplatin treatment using a xenograft tumor model, presenting the first *in vivo* evidence for the anti-cancer application of this compound. To propel future development of MASTL targeting in cancer, characterization of the next generation of MASTL inhibitors with better potency and specificity is a crucial task (Kim et al., 2020; Kang et al., 2021). On the other hand, better understanding of how MASTL functions in cell proliferation, in DNA damage responses, and in oncogenic signaling, will provide necessary guidance for the clinical applications of MASTL targeting.

## Data Availability

The raw data supporting the conclusions of this article will be made available by the authors, without undue reservation.
